# Performance of a TthPrimPol-based whole genome amplification kit for copy number alteration detection using massively parallel sequencing

**DOI:** 10.1038/srep31825

**Published:** 2016-08-22

**Authors:** Lieselot Deleye, Dieter De Coninck, Annelies Dheedene, Petra De Sutter, Björn Menten, Dieter Deforce, Filip Van Nieuwerburgh

**Affiliations:** 1 Laboratory of Pharmaceutical Biotechnology, Ghent University, Ottergemsesteenweg 460, 9000 Ghent, Belgium; 2 Center for Medical Genetics, Ghent University, De Pintelaan 185, 9000 Ghent, Belgium; 3 Department for Reproductive Medicine, Ghent University Hospital, De Pintelaan 185, 9000 Ghent, Belgium

## Abstract

Starting from only a few cells, current whole genome amplification (WGA) methods provide enough DNA to perform massively parallel sequencing (MPS). Unfortunately, all current WGA methods introduce representation bias which limits detection of copy number aberrations (CNAs) smaller than 3 Mb. A recent WGA method, called TruePrime single cell WGA, uses a recently discovered DNA primase, TthPrimPol, instead of artificial primers to initiate DNA amplification. This method could lead to a lower representation bias, and consequently to a better detection of CNAs. The enzyme requires no complementarity and thus should generate random primers, equally distributed across the genome. The performance of TruePrime WGA was assessed for aneuploidy screening and CNA analysis after MPS, starting from 1, 3 or 5 cells. Although the method looks promising, the single cell TruePrime WGA kit v1 is not suited for high resolution CNA detection after MPS because too much representation bias is introduced.

Several whole genome amplification (WGA) methods exist to amplify DNA extracted from a limited number of cells, yielding the necessary amount of DNA required to perform massively parallel sequencing (MPS)^1^,^2^. The different WGA methods each have their advantages and disadvantages in terms of genome coverage, representation bias, error rates, yield and robustness. The most appropriate method should be selected based on its intended application. A recent study suggests that multiple displacement amplification (MDA) methods are better suited for single nucleotide polymorphism (SNP) detection while PCR-based methods are the better option for copy number aberration (CNA) detection^1^. MDA methods use the high-fidelity phi29 polymerase, leading to less nucleotide errors in the amplified sequences, while PCR-based methods tend to give a more balanced genomic amplification. Recently, we compared two state-of-the-art PCR-based WGA methods to study their applicability for CNA detection using MPS^3^. In that study, Picoplex/SurePlex (Rubicon Genomics Inc., MI 48108, USA/BlueGnome Ltd., Mill Court, Great Shelford, Cambridge, UK) proved to be more suitable for CNA detection compared to Multiple Annealing and Looping Based Amplification Cycles (MALBAC) (Yikon Genomics, Beijing, China): MALBAC amplified samples showed a less uniform read distribution across the genome (i.e. more representation bias), leading to more false positive and false negative CNA detections. SurePlex amplified samples lead to accurate detection of CNA with a resolution of 3 Mb. In another study, SurePlex WGA proved its efficient amplification of DNA from 4–6 blastocyst cells for downstream MPS with a reliable detection of chromosomal aberrations down to 3 Mb^4^. Nevertheless, results show that the WGA representation bias is still a limiting factor in achieving higher resolution copy number profiles when starting from a single or a limited number of cells^4^. In order not to call over- or underamplified regions as CNAs, the read counts need to be averaged out in genomics windows of at least 0.5 Mb^4^, leading to a 3 Mb resolution for CNA detection (see also Methods section). With less representation bias, smaller windows and a higher resolution could be used. Accurate detection of CNAs from amplified DNA is of importance for applications such as pre-implantation genetic diagnosis (PGD) in which day-5 embryos are screened for CNA using of 3–7 trophectoderm cells^4^. Cell-based liquid biopsy both in cancer and prenatal diagnosis, is another emerging field where accurate, high resolution CNA detection starting from a limited number of cells is invaluable.

A WGA method called TruePrime single cell WGA (Sygnis, Heidelberg, Germany) uses a DNA primase, TthPrimPol, which synthesizes primers for Phi29 DNA polymerase, so that no artificial primers need to be added to the reaction^5^. After primer synthesis by TthPrimPol, Phi29 polymerase performs polymerization and strand displacement as in a classical MDA. The non-artificial primers, which could lead to a lower representation bias, combined with the high-fidelity of Phi29, could theoretically lead to an ideal WGA method.

The goal of this study was to assess the performance of TruePrime WGA for aneuploidy screening and high resolution copy number analysis, starting from a limited number of cells, using MPS. The variability in distribution of the reads across the genome and the ability to correctly detect chromosomal aneuploidies and large CNAs was assessed and the results were compared to a study from Deleye *et al.*^3^ in which the performance of Picoplex/SurePlex and MALBAC WGA was studied in a similar setting.

## Material and Methods

### Experimental design

**Figure 1:**
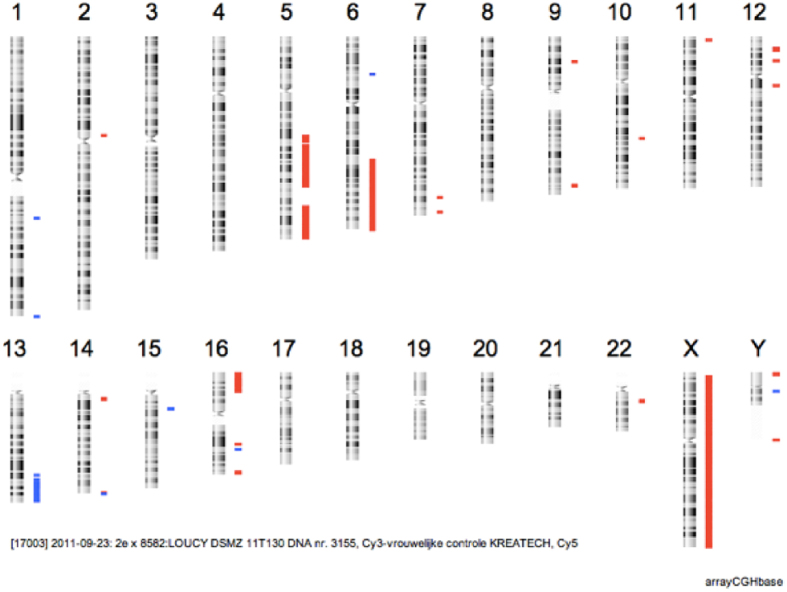
180 K arrayCGH of genomic DNA from the female LOUCY cell line. This profile shows all CNAs detected in the female LOUCY cell line up to a resolution of 50 kb. Red bars indicate deletions and blue bars indicate insertions. The deletions in chromosomes X, 5, 6, and 16 and duplication in chromosome 13, all with a size of ≥3 Mb, were the CNAs expected to be detected by the sequencing results.

**Figure 2:**
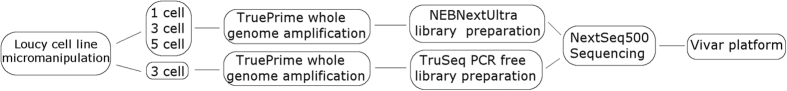
Experimental design. Schematic overview of the experimental design of the study. Samples containing 1, 3 and 5 cells were taken from the LOUCY cell line using micromanipulation. Next, the DNA of these individual samples was amplified with the TruePrime WGA kit. The WGA product of 1, 3 and 5 cell samples was used to perform an Illumina library preparation with enrichment PCR. In parallel, WGA product of 3 cell samples was used to perform a PCR-free Illumina library preparation. This experiment was done in triplicate for all sample types.

This study was performed on cells derived from the female LOUCY cell line (DSMZ, ACC394)^6^. A reference 180 K arrayCGH profile (Agilent Technologies) was obtained from unamplified genomic DNA from this cell line (Fig. 1). According to the 180 K arrayCGH profile, the following chromosomal aneuploidies and CNAs were called within the resolution range (≥3 Mb) of the subsequent sequencing results: a deletion of an entire X-chromosome, a distal deletion of ±72 Mb on 5q21.3q35.3, a distal deletion of ±45Mb on 6q22.31q27, a ±3Mb duplication of 13q33-q33.3 and deletions of respectively 13 Mb and 3 Mb on 16p13.3-p13.12 and 16q24.2q24.3. These CNAs were used as a reference for comparison with the MPS analyses. The cell line was grown in suspension which allowed isolation of individual cells. Samples consisting of 1, 3 or 5 cells, in triplicate, were collected using micromanipulation. These samples were used to perform TruePrime WGA, Illumina library preparation with enrichment PCR and sequencing. In parallel, triplicate samples consisting of 3 cells were subjected to TruePrime WGA, PCR-free Illumina library preparation and sequencing to comparatively study the effect of enrichment PCR on representation bias (Fig. 2).

A negative control was used during WGA to control for contamination of the samples. A sample containing 26 pg high quality DNA was used as a positive control during WGA. This positive control was performed to check the yield of the WGA procedure on a sample with high quality input DNA, excluding possible suboptimal conditions due to the cell manipulation and extraction steps. The positive control serves as reference of the optimal WGA product yield and could be used as a troubleshooting tool in case the cell samples would yield no (or low) amounts of WGA product. Library preparation, sequencing and data analysis were performed in parallel with other similar samples (routine samples amplified with SurePlex WGA, not belonging to present study) to rule out failing steps after WGA amplification.

### Growth and isolation of cells

The cells were grown in Rose Roswell Park Memorial Institute (RPMI-1640) medium (Life Technologies, Carlsbad, USA), supplemented with 10% fetal bovine serum (Life Technologies, Carlsbad, USA). For optimal growth, they were kept at a temperature of 37 °C and a 5% CO_2_ level. A known amount of cells was isolated with an ergonomic denuding handle from STRIPPER (Origio, Måløv, Denmark) and MXL3-100 needles with a diameter of 100 μm (Origio, Måløv, Denmark). A serial dilution with sterile phosphate buffered saline (PBS) (Life Technologies, Carlsbad, USA) spots was performed on a Petri dish (5.5 cm) under an Axiovert 25 light microscope (Zeiss, Jena, Germany), until the desired amount of cells for isolation was obtained. All cells were collected in a maximum volume of 2.5 μl. All samples were snap frozen in liquid N_2_, immediately after collection.

### TruePrime WGA

Cell lysis and amplification was performed using the TruePrime Single cell WGA kit (Sygnis, Heidelberg, Germany), following manufacturer’s instructions. As a positive control, 1 μl of 26 pg/μl single-source male control DNA (NIST 2391C Component C DNA) was used. As a negative control, 1 μl of PBS was used as input material. All samples were purified according to the manufacturer’s protocol of the Genomic DNA Clean & Concentrator kit (version 1.0.0, Zymo Research, Irvine, USA) with 5X binding buffer. Concentration was measured using Qubit dsDNA High Sensitivity Assay kit (Life Technologies, Carlsbad, USA). The quality of the different samples was assessed with the Agilent 12000 (12K) DNA Assay kit (Bioanalyser, Agilent Technologies, California, USA).

### Illumina library preparation

One hundred ng of the WGA product was fragmented to an average size distribution of 200 bp with the S2 Focused Ultrasonicator with Adaptive Focused Acoustics (AFA) technology (Covaris, Woburn, USA). All samples were diluted in 1/5X Tris-EDTA buffer (TE-buffer; stock 1X) to a volume of 130 μl in microTUBES (Covaris, Woburn, USA). The programmed guidelines for fragmentation to 200 bp were followed (Duty cycle of 10%, Intensity of 5 and 200 cycles/burst), but the fragmentation time was prolonged to 190s based on previous experience.

Subsequently, libraries of the fragmented samples were created using NEBNext Ultra DNA Library Prep (PCR 1.4A, New England Biolabs, Ipswich, USA), following manufacturer’s protocol, with the exception of some minor modifications. After incubation with the USER enzyme, a DNA purification step (Zymo Genomic DNA Clean & Concentrator) was included before the size selection step. Size selection was performed with the E-Gel iBase Power system (Invitrogen) using an E-gel EX 2% agarose gel and a 1 kb Plus DNA ladder (Thermo Fisher Scientific, Waltham, USA). For all samples, fragments with a size of ~300 bp were cut from the gel, and DNA was recovered using the Zymoclean gel DNA recovery kit (Zymo Research). The size selected DNA samples were then subjected to an enrichment PCR using NEBNext Multiplex Oligos for Illumina (Index Primers Set 1 and 2 version 3.0) according to the protocol. tRNA was added to the reaction to minimize the loss of DNA via tube interaction. The quality of the different libraries was assessed with the Agilent High-Sensitivity DNA kit (Bioanalyser, Agilent Technologies, California, USA).

The PCR-free libraries were created entirely according to the TruSeq DNA PCR-free HT sample preparation kit (Illumina), which does not require an enrichment PCR. For each sample, 1 μg of WGA product was used as input. From here onwards, these samples will be referred to as ‘PCR-free’ samples.

Before sequencing the samples, a library quantification was performed using a Sequencing Library qPCR Quantification kit (Illumina, San Diego, USA) to quantify the sequenceable DNA fragments containing the correct adapters. The control template used for the standard curve was a PhiX control library (10 nM). The libraries from the different samples were equimolarly pooled, denatured and diluted to a final loading concentration of 2.1 pM for sequencing.

Finally, single-end index 75 bp sequencing was performed on a high-output flowcell on a NextSeq500 (Illumina, California, USA).

### Data analysis

Fastq files of the samples were automatically analyzed using the Vivar software^6^,^7^. The Vivar software performs CNA detection using the QDNAseq algorithm^8^. After removal of poorly mapped reads, this algorithm normalizes the number of reads mapped in non-overlapping, fixed size windows for bias in GC-content and mappability simultaneously. In addition, QDNAseq excludes anomalous genetic regions from the analysis making use of a “blacklist” based on information from the ENCODE Project Consortium^9^. The blacklist contains chromosomal regions with known repeat elements, such as satellites, centromeres, and telomeres. After GC-content and mappability normalization, read counts were median-normalized by dividing the number of reads in each window by the median number of reads across all windows. As chromosomal aberrations were assumed to be rare, the median number of reads across all windows is a fair estimate of the expected number of reads per window for a perfectly diploid genome. As such, the median-normalized read counts represent a measure for the deviation from diploidy for each window and a copy number (CN) estimate is calculated using following formula: CN = 2(read count/median read count). Then, a circular binary segmentation (CBS) algorithm was applied which detects breakpoints between the windows, and groups them between breakpoints into larger contiguous regions with an equal CN. To this end, the CBS algorithm starts with the whole chromosome and segments it recursively by testing for change-points between such regions. The two-sample t-statistic is then applied to compare the mean of the read counts of the windows contained in one segment to the read counts of the windows in its adjourning segment. The mean of read counts of the windows contained in the segments are used as an estimator of the copy number of the whole segment. After this segmentation, CNAs are called when the segment’s log_2_(CN/2) surpasses a threshold of +/−0.35, corresponding to a CN greater than 2.55 or less than 1.57. These thresholds were chosen based on literature review and own experience^10^. From previous experience, a window size of 1 Mb should be ideal to detect chromosomal aberrations of 3 Mb and bigger^4^. Analyzed data was visualized as line plots, in which windows are ordered along the x-axis by their genomic positions, and the y-axis shows the median normalized log_2_ transformed read counts, i.e. the log_2_(CN/2). Chromosomes are identified along the x-axis by an alternating white and blue background color. Each dot on the profiles represents a different window and the horizontal lines refer to the segments. Each genomic profile was manually checked for aberrations.

### Read distribution analysis

The read distribution in the samples was further analyzed using Integrative Genomics Viewer (IGV)^11^. The BAM file of a sample was uploaded and hg19 was selected as the human reference genome. Next, a region was selected to zoom-in until the individual reads were visible. The selected regions covered a 1 Mb window or one dot on a line profile on Vivar. Windows with a very high, low or average CN were investigated.

### Data dereplication

Reads of which the start is mapped to the same genome position were dereplicated from the BAM files (containing the mapped reads) using a Python script. From a group of reads with an identical start position, only one is kept. Reads with an identical sequence are thus also dereplicated.

### Sensitivity and positive predictive value

The sensitivity was defined as the number of true positive calls divided by all CNA present in the reference (See details in Experimental design section). The positive predictive value (PPV) was defined as the number of true positive calls divided by the total number of calls (true positive + false positive).

True positives are CNA calls that refer to a chromosome region that differs at most 10% in size in from the corresponding CNA in the reference: When e.g. a complete chromosome is called, and only a part of that chromosome has a CNA in the reference, the call counts as a false positive.

### Statistical analysis of the read count variance

For each sample, the read count variance observed between the windows across the whole genome is calculated using formula (1): 
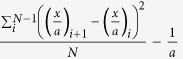
 where ‘*N’* is the number of windows, ‘*x*_*i*_ ’ the read count in window *i,* ‘*x*_*i*__+*1*_’ the read count in the next window *i* + *1* and *‘a’* the average of the read counts in all windows^8^,^12^. In this formula, the read count in each window is scaled by factor ‘a’, normalizing the result for the total number of reads that was sequenced for the sample. In the formula, 1/a is subtracted from the sum to account for the variance that is due to random generation of the reads across the genome during sequencing (described by the Poisson distribution) so that the result reflects only the variance that is due to sample processing (which includes WGA). This measure was calculated for each sample. A Welch’s t-test for unequal variances was performed to compare all 12 (3 repeats each of 1-, 3- and 5-cell samples +3 3-cell enrichment PCR-free samples) TruePrime amplified samples *versus* all 12 analogous previously reported Sureplex amplified samples^3^. A similar analysis was performed using the dereplicated TruePrime data. P-values smaller than 0.05 were considered statistically significant.

## Results

### Sample quality and yield after WGA

All samples had a similar electropherogram (EPG) on a 12 K chip after WGA (Supplementary File 1). The DNA fragments resulting from amplification had an average length of 5 kb. The negative control showed a flat EPG and the positive control an EPG similar to 5 cell samples.

The WGA yield increased with increased amounts of DNA input material. The average output after WGA on 1, 3 and 5 cells was respectively 1684 ± 120.4 ng (mean ± standard deviation), 2470 ± 88.6 ng and 2629 ± 38.7 ng. The positive control yielded an output of 2387 ng, while the negative control had an output of 22 ng. This small amount of WGA product in the negative control, is most probably the result of primer-dimer formation and is normal in negative control WGA products.

### Sequencing run statistics

The quality control parameters of the sequencing libraries were flawless. Supplementary File 2 shows the Agilent Bioanalyser 2100 results of the individual libraries (A), as well as the qPCR library quantification results (B). The Bioanalyser results show library size distributions within the range of 200–1000 bp, which is suitable for clustering on the Illumina flow cell. The qPCR results show a variable but adequate amount of sequenceable library within the expected yield range for NEBNext Ultra library preparations. Based on these results the libraries were equimolarly pooled. The overall sequencing run quality was good: a Q30 of 90.1 ± 0.5, a density of 269.25 ± 1.3 K/mm^2^ and a full width at half maximum (FWHM) of 3. On average 34 million passed filter reads were obtained per sample, corresponding with an average read depth of 0.8 per sample if all reads could be mapped. This number of reads is a multitude of what is needed to perform CNA detection at a 3 Mb resolution^4^. Supplementary File 3 shows an elaborate sequencing run statistics quality control report. On average 96.3% of these reads mapped to the reference human genome, of which 97.8% mapped uniquely to the reference. The actual average read depth and coverage, calculated after mapping, are 0.77 and 0.04 respectively. This reflects a completely uneven distribution of the reads over the genome. The standard deviation of the read depth across the genome in individual samples is indeed very high. Raw sequencing data (FASTQ files) has been deposited in the NCBI Sequence Read Archive (SRA) under project accession number PRJNA318625.

### Visualization of the data on Vivar

**Figure 3:**
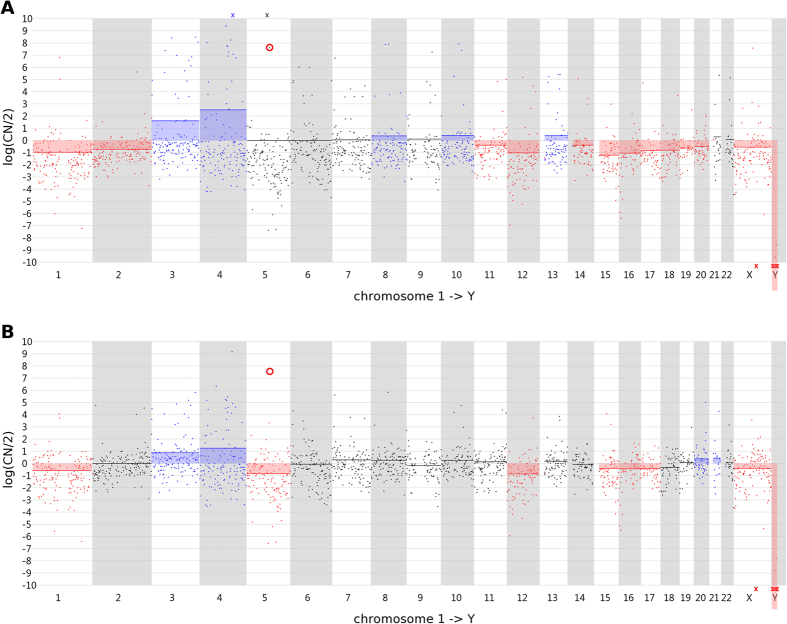
CNA profiles before and after dereplication of the data. (**A**) A line profile of sample ‘3 cells, replicate 1’ before dereplication, including the ‘outlier’ windows such as the one in the red circle. (**B**) The same line profile after dereplication shows some improvement. High outliers such as the one in the red circle are no longer present. However, windows with a very low CN were still observed and the CNA profile is still littered with deletions and insertions. The blue color indicates a duplication or trisomy, whereas the red color indicates a deletion or monosomy.

The results of the data analysis workflow were plotted on a graphical line profile using the Vivar software. A uniform distribution of the reads over the windows across the genome (except for the parts with a CNA) would be the preferred result. However, the read counts per window from our samples were extremely irregular across the genome (Fig. 3A). Although all profiles were similar within and between the 1, 3 and 5-cell samples, the exact regions showing over- or under-representation were different from sample to sample. The line plots of all samples are available in Supplementary File 4. A consequence of the uneven distribution of the reads is that a lot of window segments had a CN greater than 2.55 or less than 1.57, leading to falsely called CNA in regions where the LOUCY genome is known to be diploid. The expected chromosomal abnormalities could also not be deduced from the line profiles. The sensitivity for TruePrime amplified samples was 18.5% ± 5.6% (mean ± standard deviation), whereas for SurePlex amplified samples this was 94.4% ± 11.8%2. Except for the deletion of chromosome X, which was called correctly in all samples, only one other CNA was called correctly in one of the samples. Most often, complete chromosome aneuploidies were called where only chromosomal parts have a CNA in the reference. Both WGA methods, also resulted in a significantly different PPV of 8.43% ± 3.16% and 77.7% ± 11.8% for TruePrime and SurePlex respectively. The distinctive difference between the PPV of TruePrime and SurePlex is due to the presence of many more false positives after TruePrime WGA than after SurePelex WGA.

### Statistical analysis of the read count variance

**Figure 4:**
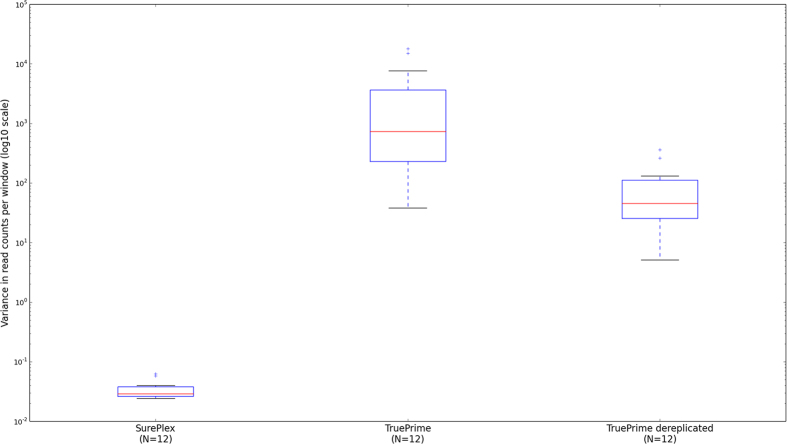
Boxplots of the variances in read counts per window across the genome. Boxplots are shown for all TruePrime samples, the dereplicated TruePrime samples and previously reported SurePlex samples^3^.

The variance of the read counts per window across the genome for the current TruePrime samples is a lot higher compared to the variance in the previously analyzed SurePlex samples^3^ (p = 0.015; Fig. 4). A similar observation was made when comparing the dereplicated Trueprime data with the Sureplex data (p = 0.052; Fig. 4). A table containing the calculated variances for SurePlex and Trueprime amplified samples can be found in Supplementary File 5.

### Visualization of read alignment in IGV

**Figure 5:**
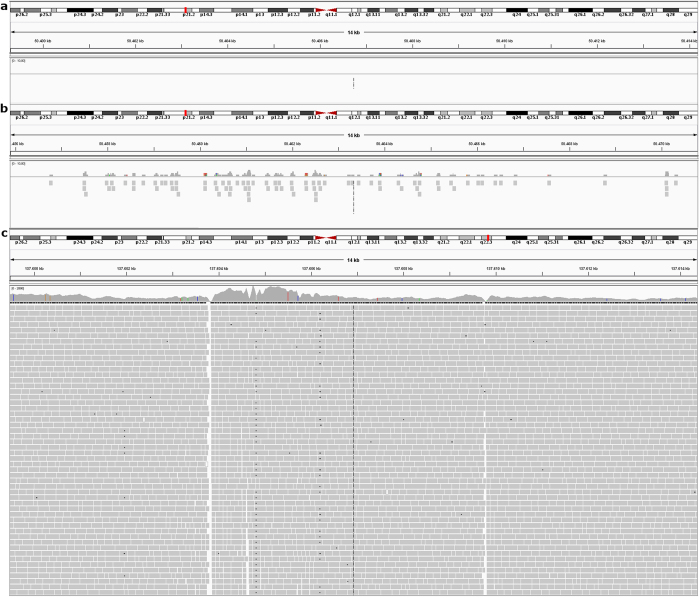
Read alignment in IGV. A print-screen is shown for 3 different 14 kb regions on chromosome 3 of sample ‘5 cells, replicate 2’. The read alignment to the reference genome hg19 is illustrated for these regions. (**a**) A region in a window close to the baseline (CN = 1.9) with a complete lack of reads. (**b**) A small clustering of reads in another region of this window. (**c**) A massive buildup of reads in a 48 kb wide region in an ‘outlier’ window with a CN of 89.9. Only a part of this buildup is shown here.

To explain why some windows had extremely high read counts, a better insight into the read distribution within these windows was needed. Two windows were analyzed using IGV, one having a CN close to 2 (CN = 1.9) and one ‘outlier’ (CN = 89.9) on chromosome 3 of a 5-cell sample amplified with TruePrime. The first window did not show the random equal distribution of reads expected from a low-pass sequencing. A lot of regions, larger than could be expected for a random distribution of reads across the window, showed a complete lack of reads (Fig. 5a) and whereas others showed small clusters of reads (Fig. 5b). The ‘outlier’ window showed a massive buildup of reads halfway the window over a region of 48 kb (Fig. 5c).

Supplementary File 6 shows the read distribution of a few representative regions, comparing the previously studied SurePlex WGA and the currently studied TruePrime WGA. SurePlex WGA gives a much more even distribution of the reads compared to TruePrime WGA.

### Read distribution across the genome and CNA detection on dereplicated data

When sequencing at an average read depth below 1, only a minor fraction (<1%) of the reads should have the same starting position when there is no representation bias. When the number of mapped reads obtained for the individual samples (ranging between 2.2 E + 07 and 5.5 E + 07) in this study, would be randomly distributed over the genome, approximately 0.4 to 0.9% of the reads would not have a unique starting position. A lot of the representation bias introduced by TruePrime sequencing is caused by regions which are highly covered with duplicate or overlapping reads. We explored if the analysis could benefit from the removal of reads with the same starting position. Only 22% ± 12% of the originally mapped reads were left after dereplication. The dereplicated data resulted in a lower read count variability across the genome and a reduced number of positive outliers (Fig. 3b). Still, the variability is higher in the TruePrime results compared to the SurePlex results (Fig. 4). As a consequence, the CNAs could still not be correctly deduced from the profile. The sensitivity and PPV didn’t change substantially.

### Library preparation without enrichment PCR

Omitting the enrichment PCR during library preparation did not influence our results. The results showed very similar profiles to the samples that were amplified, including the large read variability and the inadequacy to detect the CNAs (Supplementary File 4). Also after dereplication, the results were similar to the enriched samples.

## Discussion

The new TruePrime WGA kit looks very promising from a theoretical point of view and should introduce less representation bias. However, during this study the advantage of this kit over other WGA methods for the detection of CNAs could not be demonstrated. A proper CNA detection using MPS was not possible on samples amplified with this kit. In a similar study from our research group, SurePlex WGA has been considered most suitable for this application^3^. The results from the current study were compared to these previously generated SurePlex WGA results.

Several factors, other than the TruePrime WGA, that could lead to representation bias were ruled out. The setup of this study was equal to the SurePlex study^3^, except for the WGA method used. The same cell line and cell isolation techniques were used. All EPG profiles of the WGA samples were similar and showed the expected pattern according to the manufacturer. No contamination was introduced during the preparation of the samples, as indicated by the flat EPG of the negative control. The WGA amplification was successful in term of yield as all samples and the positive control produced adequate amounts (>1 μg) of WGA product. Library preparation, sequencing and data analysis were performed in parallel with other similar samples (routine samples amplified with SurePlex WGA, not belonging to present study). These samples yielded the expected results with a representation bias as reported before^3^ (data not shown), ruling out that steps following the WGA are introducing the observed representation bias. The kit was stored at −20 °C, as recommended by the manufacturer, for two months. The use of a kit from an exceptional bad batch was excluded, as a second kit from a different batch led to the same results.

The yield after amplification was quite high: Compared to the SurePlex study, the lowest yield was 1.4 times higher than the lowest yield with SurePlex WGA (1684 ± 120.4 ng vs 1212.1 ± 99.9 ng). The high TruePrime WGA yields enable the use of this WGA material for multiple simultaneous applications.

The sequencing run from these samples was of high quality and the created reads were almost exclusively composed of human genomic material, as on average 96.3% mapped to the human genome of which 97.8% mapped uniquely. In this respect, the TruePrime amplified samples perform better than samples amplified with SurePlex or MALBAC. However, these mapping results disclose no information regarding the uniformity of read distribution. A high mapping rate does not implicate an equal distribution of these reads along the genome. This was already clear in the previous study where MALBAC had significantly higher mapping rates compared to SurePlex, but the read distribution for MALBAC was less uniform compared to SurePlex. Unfortunately, TruePrime WGA causes an even less uniform read distribution. The per-window read count shows extreme outliers. As a consequence of this non-uniform read distribution, the expected CNA profile for the LOUCY cell line could not be detected.

A thorough data review in IGV led to a better understanding of the read distribution within these ‘outlier’ windows. The reads of these outliers clustered together in a limited number of regions within the window. Although windows with ‘normal’ CN values did not show such clustering, the read distribution was still highly non-uniform within these windows. When low-pass sequencing is performed, reads should be uniformly distributed along the genome, with regular intervals and without clustering. A uniform, unbiased read distribution is necessary for a reliable CNA determination, because over- and underrepresentation due to the WGA will disturb the CNA determination.

In an effort to make the data more suitable for CNA detection, the outliers were filtered from the data by dereplicating reads with the same starting position from the data. Hereby, identical sequences, commonly caused by enrichment PCR, are dereplicated. Reads with the same genomic mapping position but that don’t necessarily have the same length, which could have been caused by preferential binding of the TthPrimPol primase to certain regions, are also dereplicated. After dereplication only 22% of the reads remained, highlighting the magnitude of the problem of duplicated reads in a situation were almost no reads with the same starting position were expected. Nevertheless, a highly non-uniform read distribution remained after dereplication, albeit with slight improvements. As a consequence of this non-uniform read distribution, the expected CNA profile for the LOUCY cell line could still not be detected. It is of concern that true positive duplications could also be removed when reads with the same starting position are removed. This is not the case in shallow whole genome sequencing with a coverage below 1x. When the reads would be randomly distributed over the genome, less than 1% of the reads would have the same starting position. Even in duplicated regions, very few reads with the same starting position are to be expected.

Enrichment PCR and PCR-free libraries yielded the same results, showing that the enrichment PCR during library preparation was not causing the representation bias. The amount of input DNA for the WGA was also not influencing the results, as the results for 1-, 3- and 5-cell were similar.

It is not clear what is causing the representation bias. The main difference between the TruePrime WGA and other WGA methods is the use of TthPrimPol primase instead of primers. A primase should randomly bind to the DNA, because no complementarity is required. If this primase favors some regions over others, this could lead to over- and under amplification of these regions, exactly as in our results. We however see that the overrepresented regions are different from sample to sample, suggesting that the primase is not systematically favoring the same DNA regions. Possibly, too few (random) primers are formed, leading to underrepresented regions. The ones that are formed, are heavily amplified and lead to extremely overrepresented regions. We hope that our study can provide insights that can help to improve this technology, leading to WGA with less representation bias.

## Conclusion

The single cell TruePrime WGA kit version 1 is not suited for CNA detection after MPS. Representation bias seemed to be introduced during amplification, resulting in over-and underrepresentation of genomic regions.

## Additional Information

**How to cite this article**: Deleye, L. *et al.* Performance of a TthPrimPol-based whole genome amplification kit for copy number alteration detection using massively parallel sequencing. *Sci. Rep.* **6**, 31825; doi: 10.1038/srep31825 (2016).

## Supplementary Material

Supplementary Information
