# Supplementary material for: Performance of a TthPrimPol-based whole genome amplification kit for copy number alteration detection using massively parallel sequencing
Source: Sci Rep. 2016 Aug 22;6:31825. doi: 10.1038/srep31825 (PMC4992833; doi:10.1038/srep31825)

Performance of a TthPrimPol-based whole genome amplification kit for copy number alteration detection using massively parallel sequencing

Lieselot Deleye[1], Dieter De Coninck[1], Annelies Dheedene[2], Petra De Sutter[3], Björn Menten[2], Dieter Deforce[1,#], Filip Van Nieuwerburgh[1,#,*]

[1]Laboratory of Pharmaceutical Biotechnology, Ghent University, Ottergemsesteenweg 460, 9000 Ghent, Belgium.
[2]Center for Medical Genetics, Ghent University, De Pintelaan 185, 9000 Ghent, Belgium.
[3]Department for Reproductive Medicine, Ghent University Hospital, De Pintelaan 185, 9000 Ghent, Belgium.
[*]Corresponding author (email: Filip.VanNieuwerburgh@ugent.be)
[#] These authors contributed equally

Supplementary Files

Supplementary File 1.
Agilent Bioanalyser 2100 12K electropherograms of Trueprime WGA products from all samples.

Supplementary File 2.
Agilent Bioanalyser 2100 electropherograms (A) and QPCR library quantification results (B) of the sequencing libraries.

Supplementary File 3.
Sequencing quality control report and read mapping statistics.

Supplementary File 4.
Vivar line plots of all samples.

Supplementary File 5.
Read count variance between the windows across the whole genome for TruePrime and SurePlex amplified samples.

Supplementary File 6.
Read distribution of a few representative regions, comparing the previously studied SurePlex WGA and the currently studied TruePrime WGA.

Supplementary File 1.

Agilent Bioanalyser 2100 12K electropherograms of Trueprime WGA products from all samples.

All samples have a similar profile, with an average fragment length of ± 5kb. The negative control, in which the template was substituted with PBS, the electropherogram signal is a flat line, indicating the absence of DNA amplification. The positive control showed a similar profile to the other samples. In this positive control, the template was 26pg of DNA from the 2391C Component C. The Y-as shows the fluorescence intensity, which is an indicator for the amount of DNA present. The X-as shows the fragment length in base pairs.

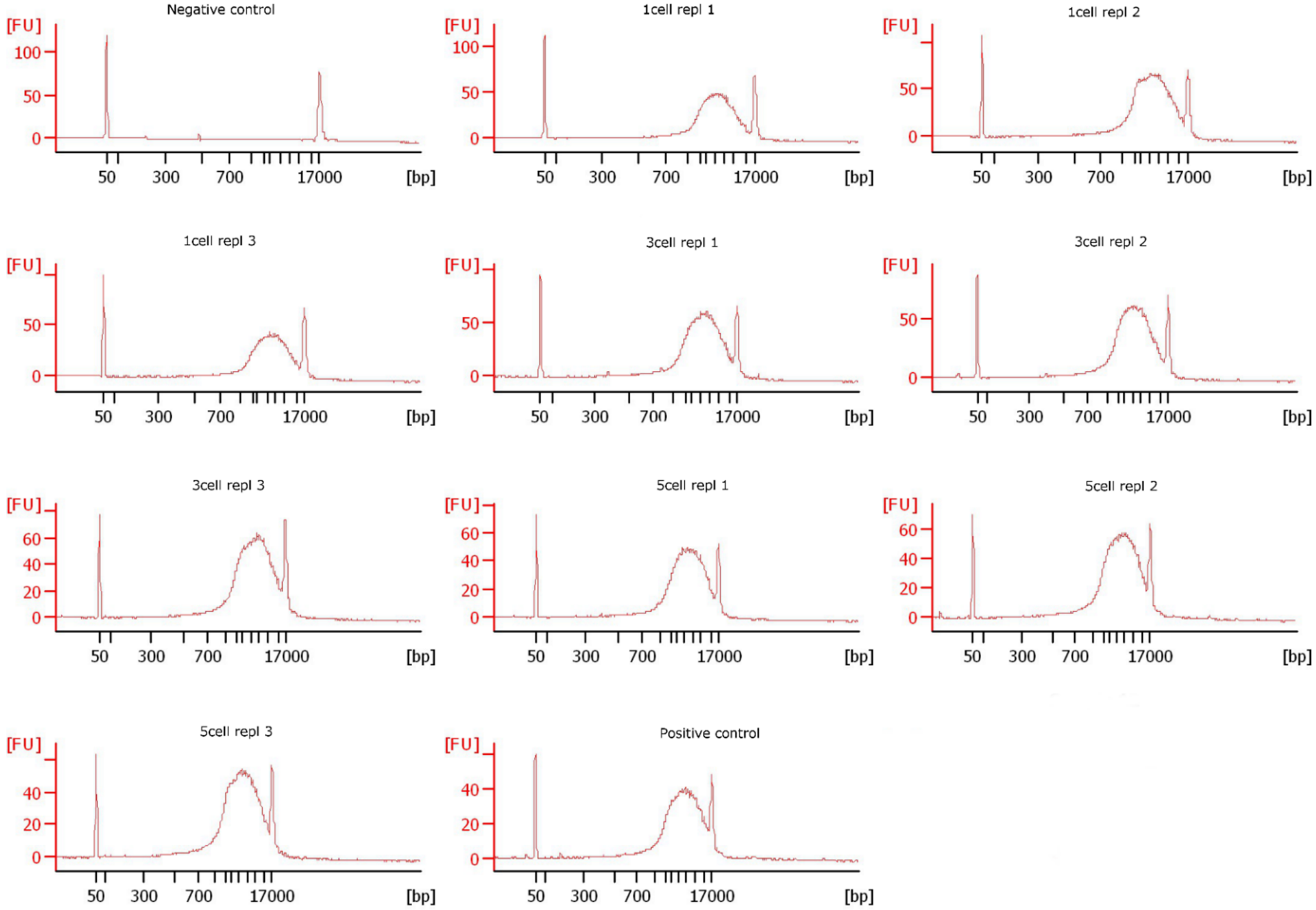

Supplementary File 2.

Agilent Bioanalyser 2100 electropherograms (A) and qPCR library quantification results (B) of the sequencing libraries.

Samples 1 to 9 were prepped using the NEBNext ultra library preparation kit. The last 3 samples were prepped using the TruSeq DNA PCR-free HT library preparation kit. Sample 5cell repl 2 shows a high primer-dimer peak, which has subsequently been removed using a 2% EX E-gel size selection.

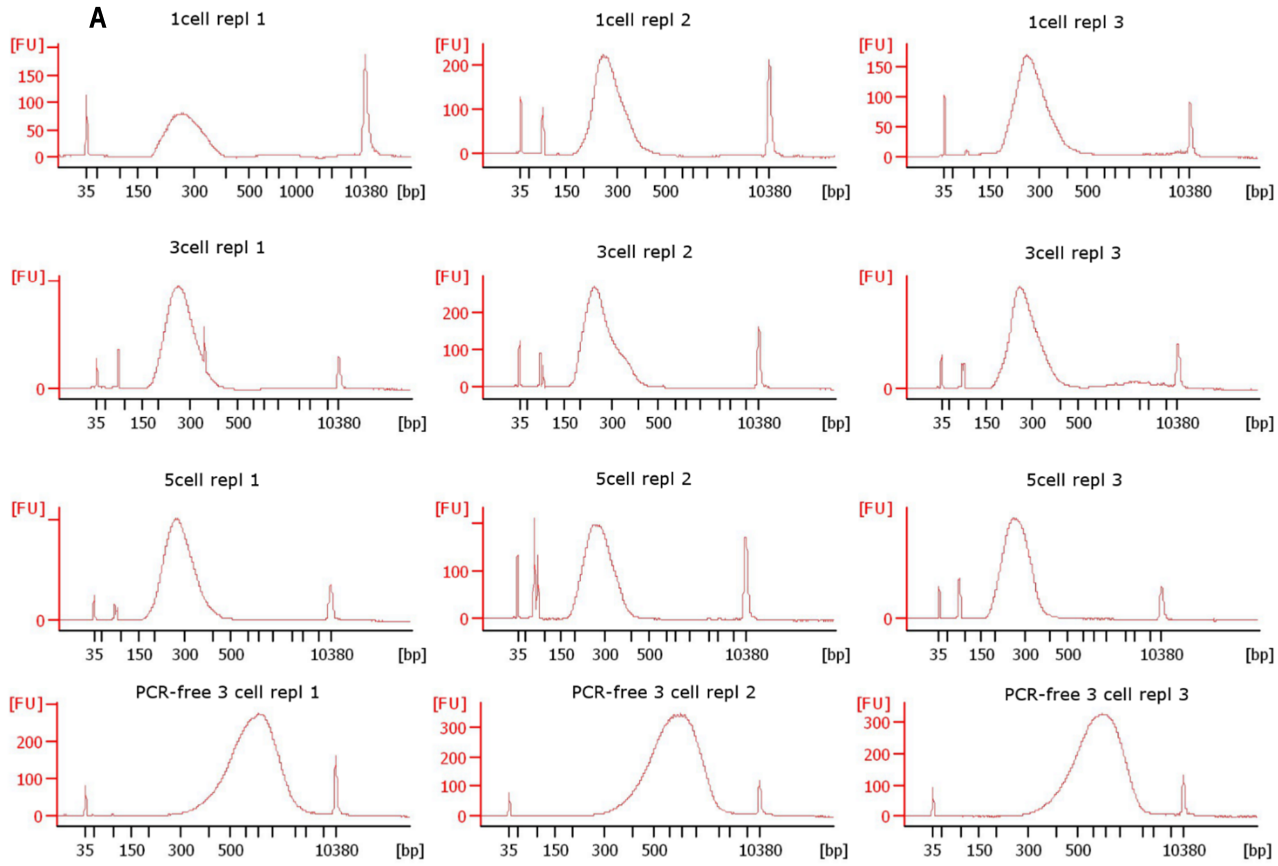

B

Samples	Undiluted library concentration (nM)
1cell repl 1	3.7
1cell repl 2	9.4
1cell repl 3	15.8
3cell repl 1	19.95
3cell repl 2	10.71
3cell repl 3	17.8
5cell repl 1	22.08
5cell repl 2	4.7
5cell repl 3	15.7
PCR-free3cell repl 1	5.8
PCR-free3cell repl 2	5.4
PCR-free3cell repl 3	6.1

Supplementary File 3.

Sequencing quality control report and read mapping statistics.

The information in the table displays the average over the 4 lanes. The run was performed with 20 samples, including 12 samples from this study and 8 samples from another (ongoing, unpublished) study.

**Density (K/mm^2)**	269.25±1.3				
**Clusters past filter (%)**	83.2±0.7				
**Reads past filter/lane (x10^6)**	145±1.8				
**Total read count past filter(x10^6)**	581.63				
**Reads/sample (x10^6)**	29.1				
**Q30 (%)**	90.1±0.5				
**Mapping statistics**	**number of past filter reads**	**number of mapped reads**	**average depth**	**standard deviation depth**	**coverage**
**1 cell replicate 1**	26,876,572	26,485,659	0.62	181.35	0.017038
**1 cells replicate 2**	21,931,292	21,690,048	0.51	38.87	0.021864
**1 cells replicate 3**	28,375,813	26,675,758	0.61	227.65	0.029697
**3 cells replicate 1**	36,862,692	36,145,552	0.84	228.08	0.034895
**3 cells replicate 2**	42,893,602	41,766,495	0.97	187.97	0.0236
**3 cells replicate 3**	30,007,070	29,259,675	0.68	134.71	0.042562
**5 cells replicate 1**	58,193,351	54,676,410	1.25	661.60	0.058997
**5 cells replicate 2**	34,894,399	33,703,063	0.79	233.71	0.071542
**5 cells replicate 3**	29,382,392	28,768,667	0.68	91.36	0.060076
**PCR-free 3 cells replicate 1**	39,486,483	37,063,877	0.85	475.55	0.042761
**PCR-free 3 cells replicate 2**	33,362,926	32,478,336	0.76	146.33	0.021173
**PCR-free 3 cells replicate 3**	29,333,156	27,753,005	0.64	257.43	0.03613

Supplementary File 4.

Vivar line plots of all samples.

<u>TruePrime 1cell repl 1</u>

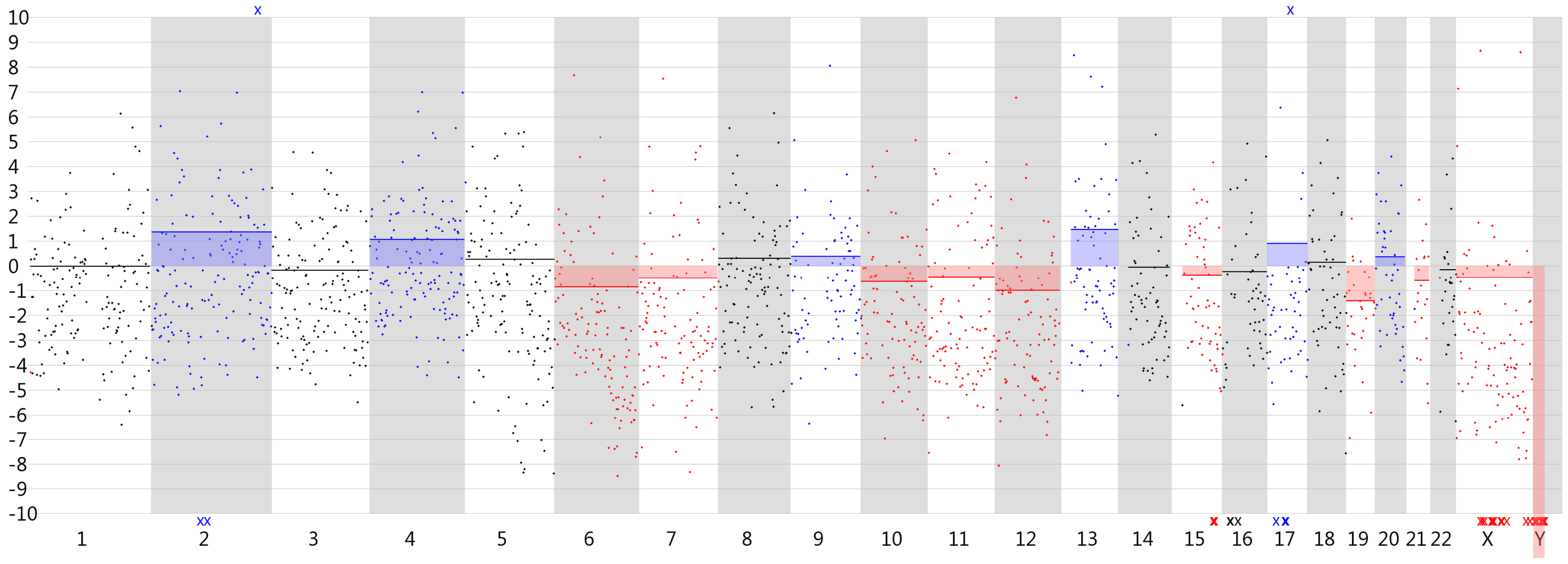

TruePrime 1cell repl 2

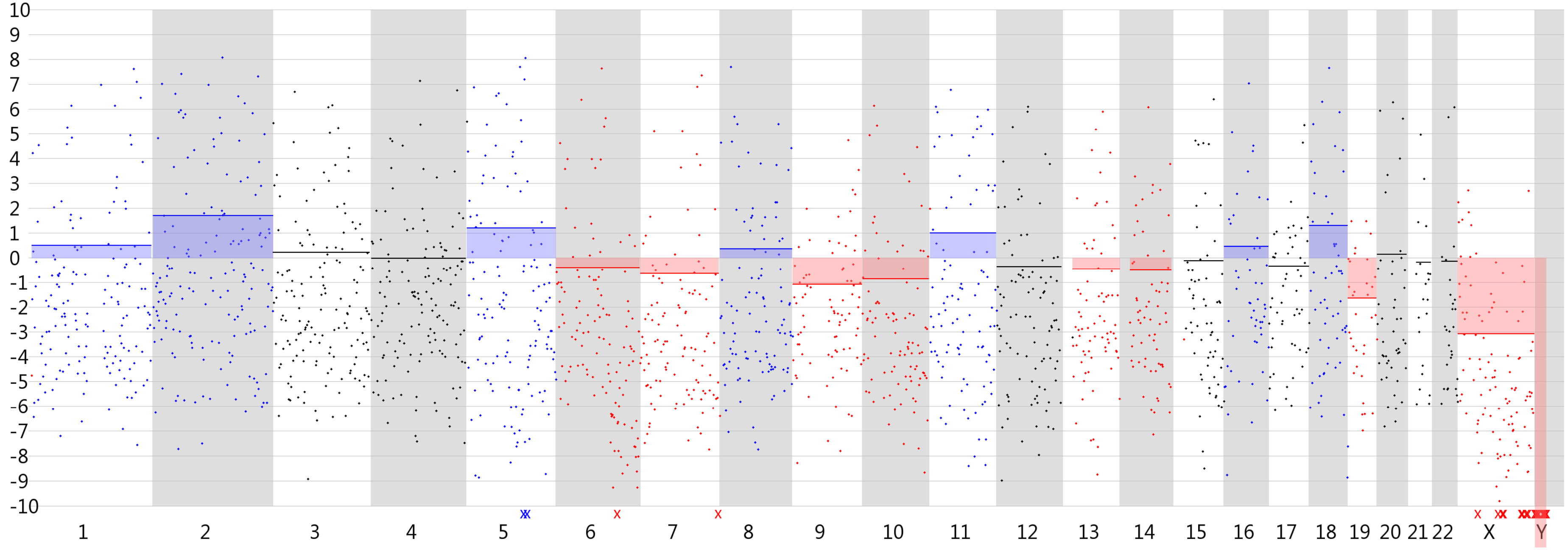

TruePrime 1cell repl 3

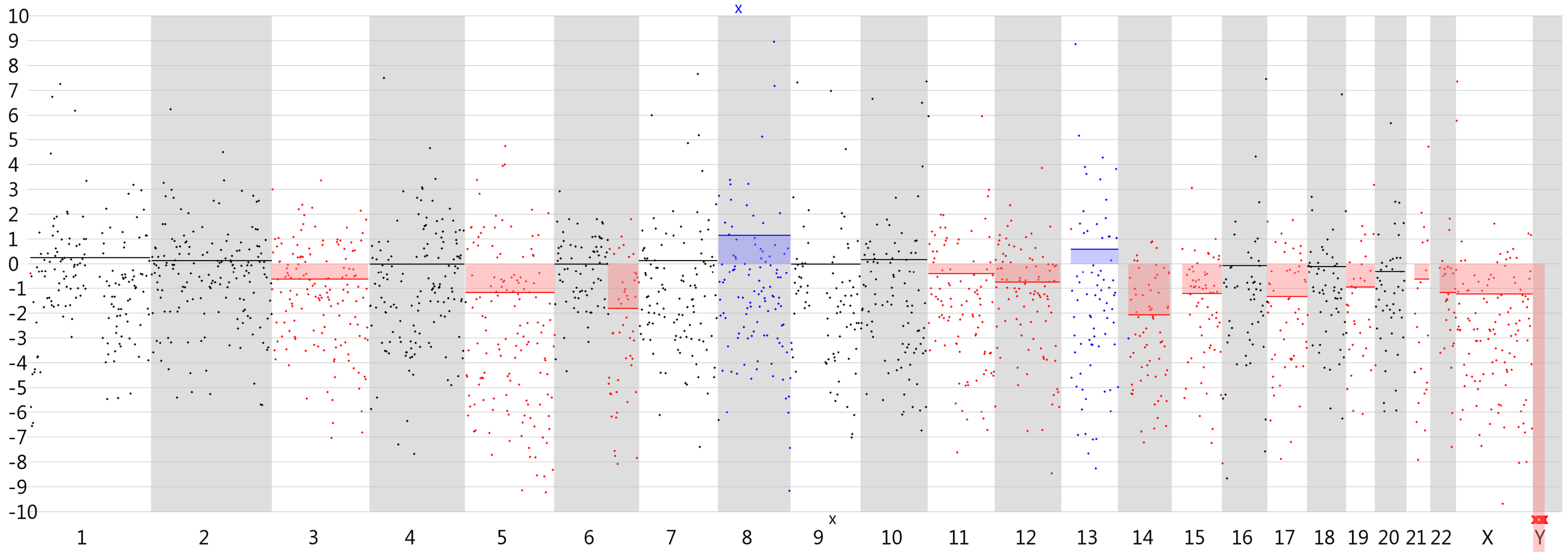

TruePrime 3cell repl 1

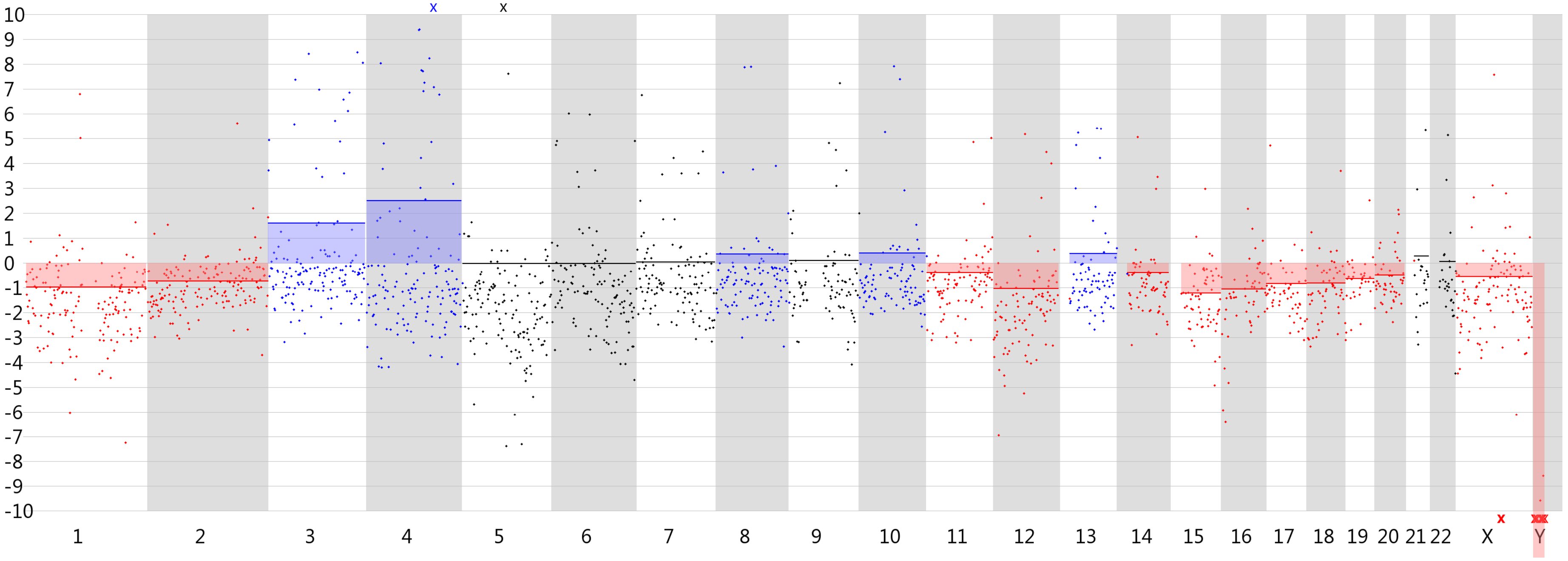

TruePrime 3cell repl 2

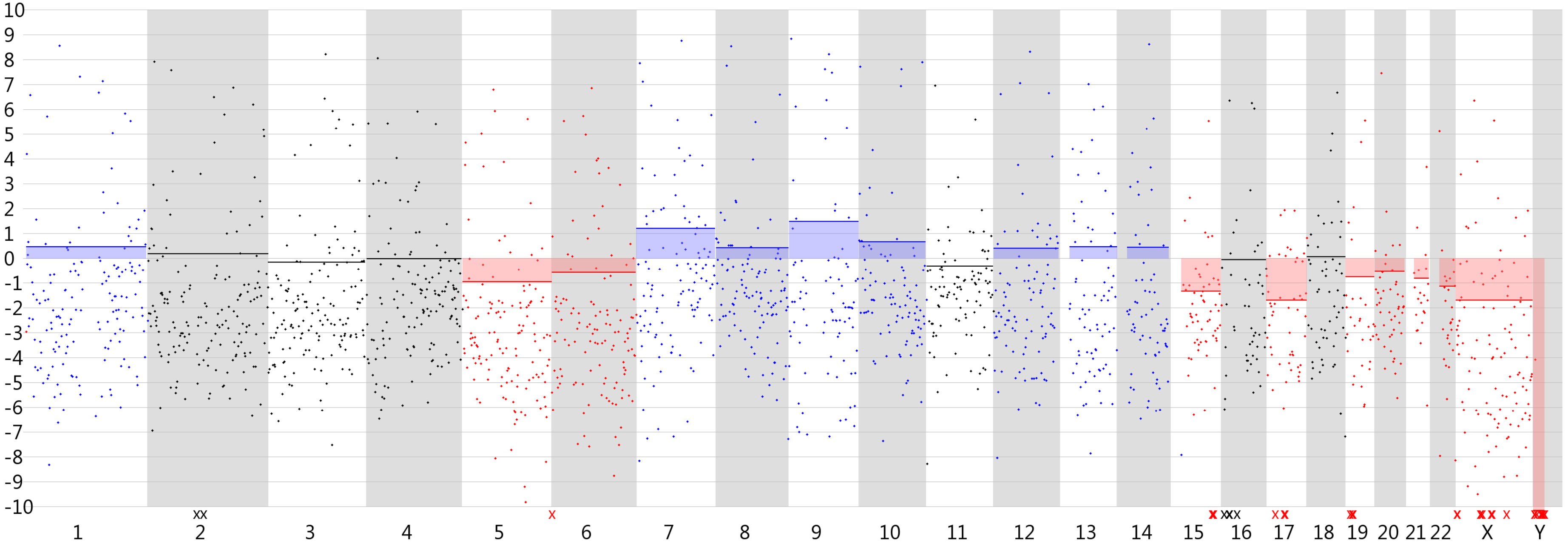

TruePrime 3cell repl 3

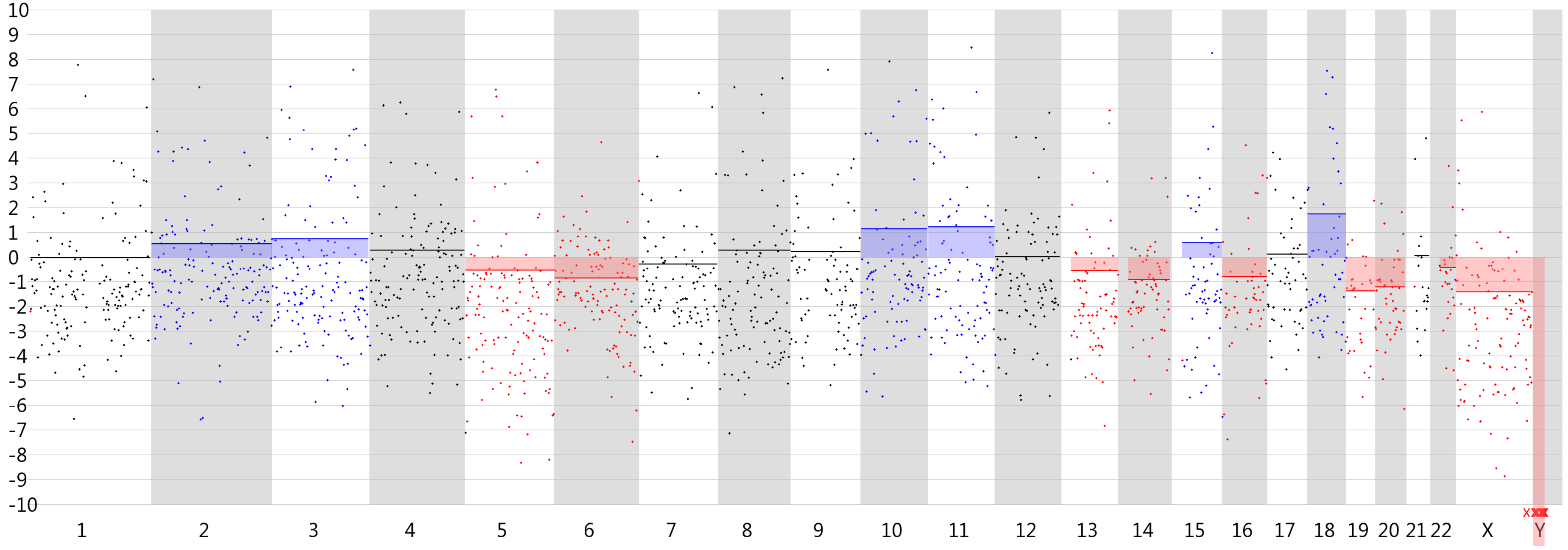

TruePrime 5cell repl 1

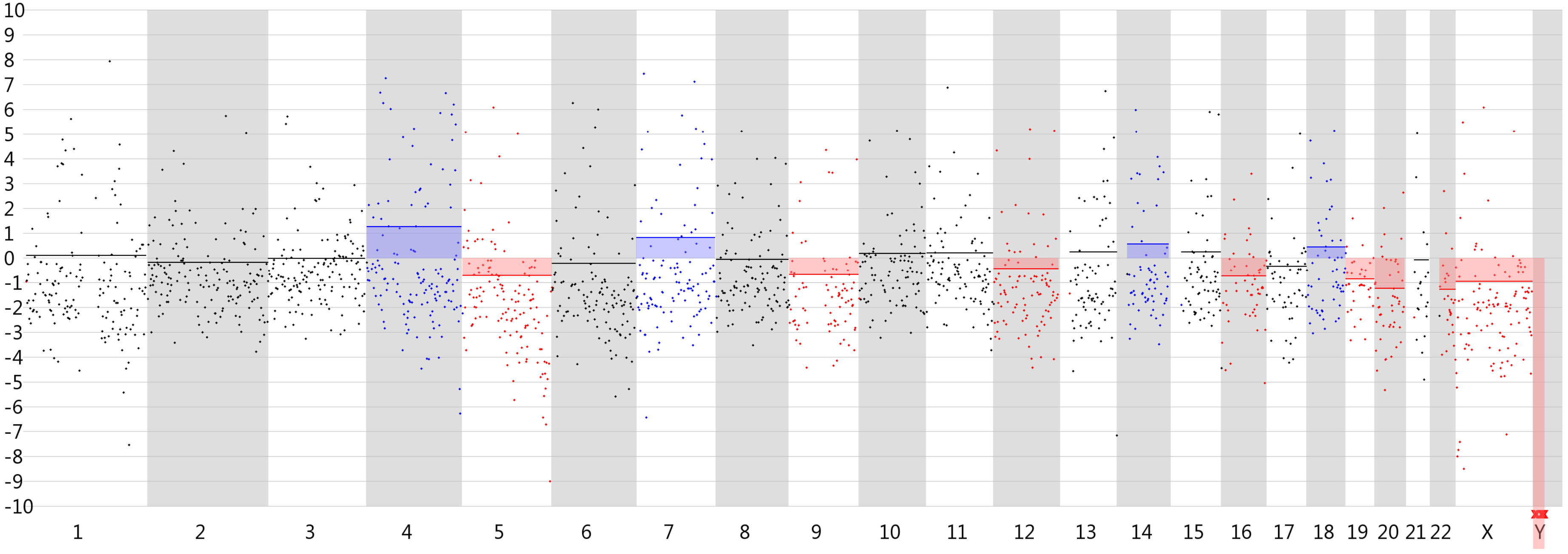

TruePrime 5cell repl 2

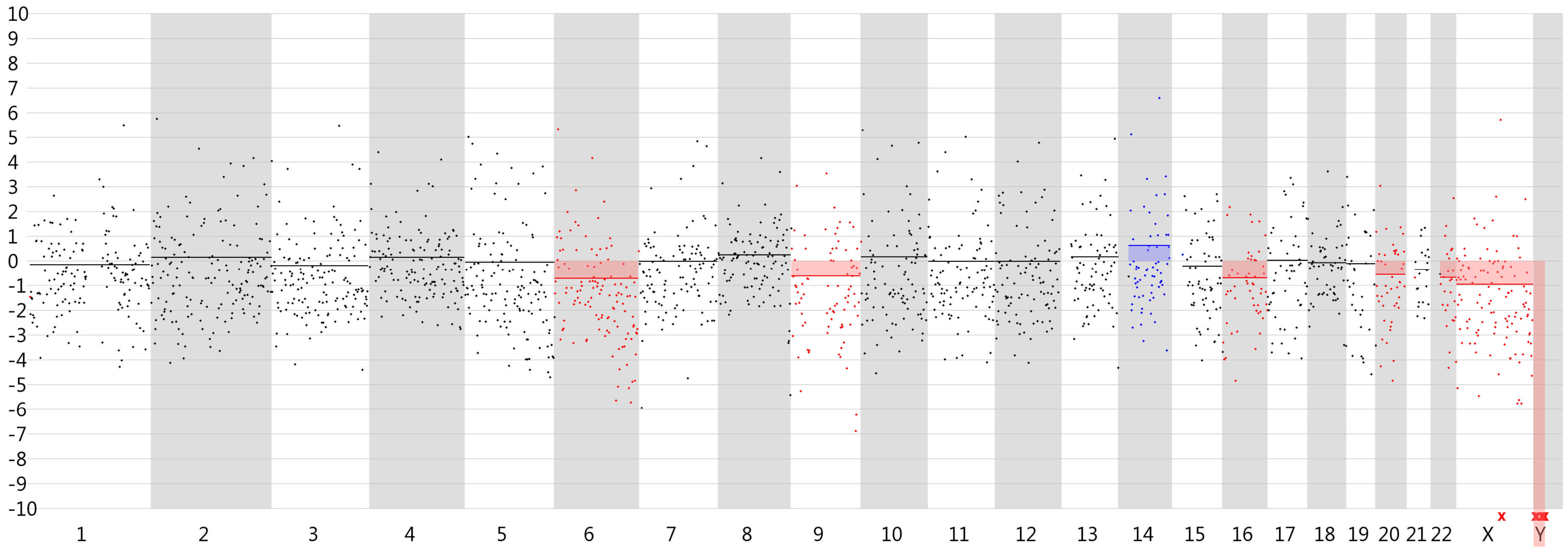

TruePrime 5cell repl 3

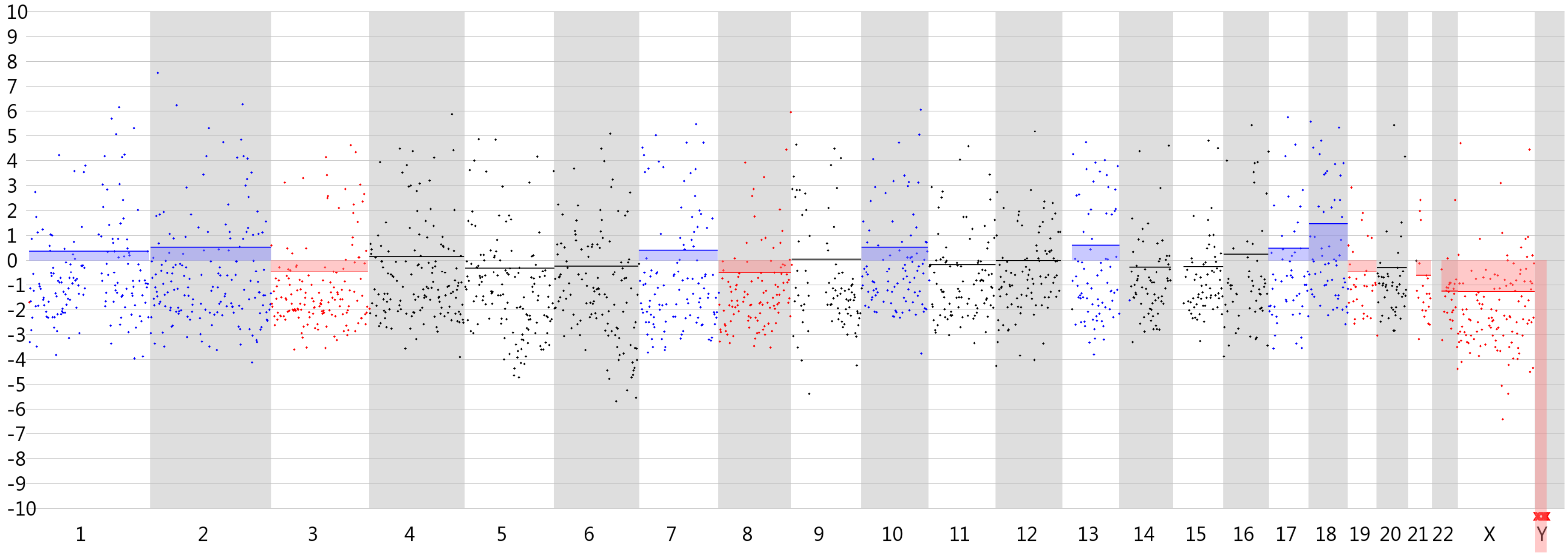

Supplementary File 5.

Read count variance between the windows across the whole genome for TruePrime and SurePlex amplified samples.

	**SurePlex**	**TruePrime**	**TruePrime dereplicated**
1cell repl 1	4.84	1.24E+20	7.96E+09
1cell repl 2	1.48	1.58E+14	5.43E+10
1cell repl 3	7.79	1.91E+18	5.94E+11
3cell repl 1	2.16	1.78E+21	9.14E+15
3cell repl 2	3.00	5.29E+15	7.36E+12
3cell repl 3	1.43	5.59E+14	2.41E+10
5cell repl 1	3.39	3.64E+13	1.67E+06
5cell repl 2	3.97	6.41E+10	6.69E+07
5cell repl 3	1.52	4.78E+12	1.05E+09
PCR-free 3cell repl 1	2.02	9.39E+10	7.35E+08
PCR-free 3cell repl 2	1.36	2.87E+19	8.14E+12
PCR-free 3cell repl 3	1.51	2.22E+15	2.26E+10

Supplementary File 6.
Read distribution of a few representative regions, comparing the previously studied SurePlex WGA and the currently studied TruePrime WGA.
a) In the SurePlex amplified sample were the reads uniformly distributed across the different windows. The TruePrime samples showed large empty regions alternated with regions of clustered reads. b) Some regions show massive clustering after TruePrime amplification.

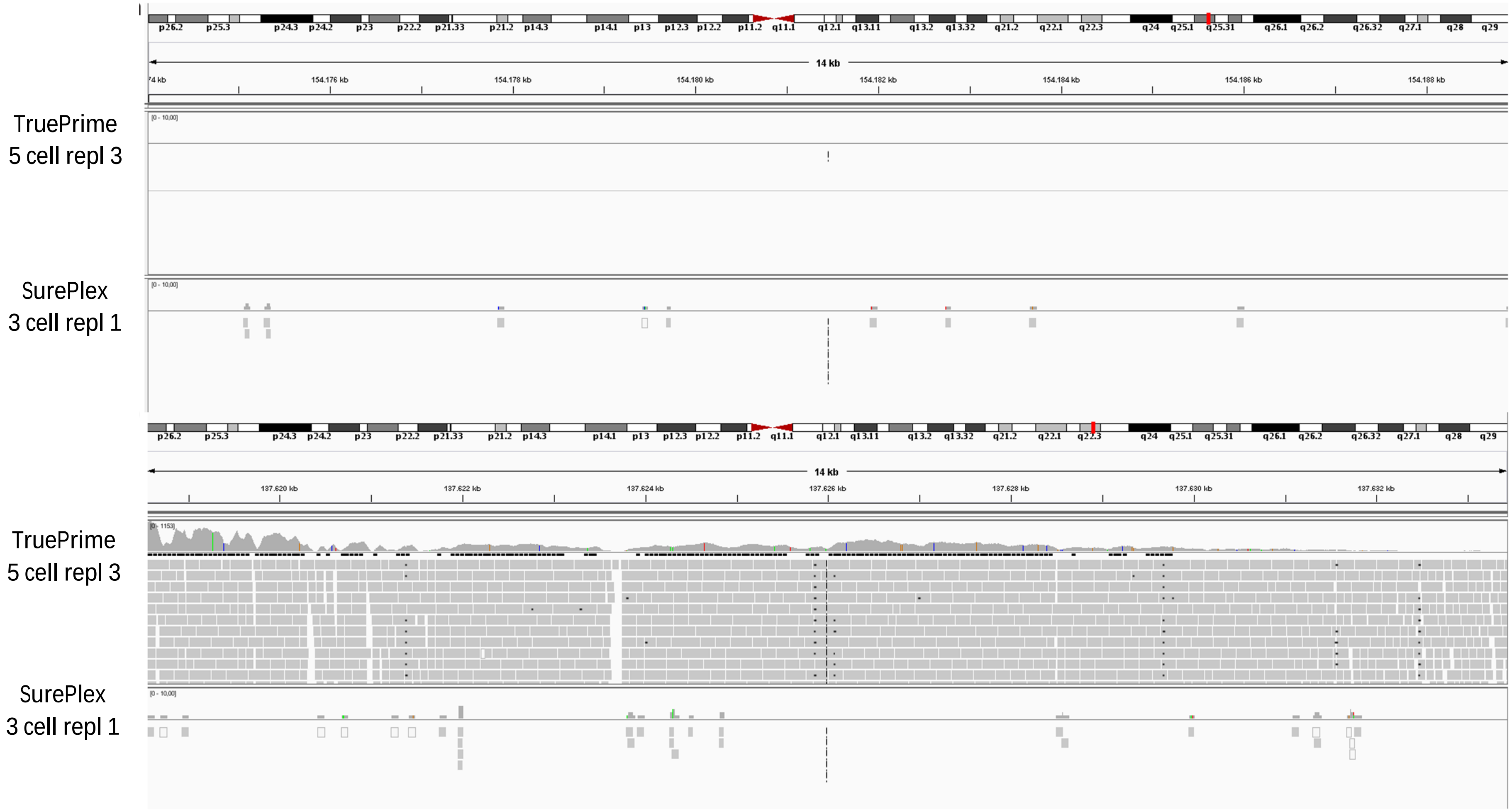

Supplement: Supplementary Information [file srep31825-s1.pdf]
